# The laboratory in the multidisciplinary diagnosis of differences or disorders of sex development (DSD): III) Biochemical and genetic markers in the 46,XYIV) Proposals for the differential diagnosis of DSD

**DOI:** 10.1515/almed-2021-0043

**Published:** 2021-07-05

**Authors:** Maria Luisa Granada, Laura Audí

**Affiliations:** Department of Clinical Biochemistry, Hospital Germans Trias i Pujol, Autonomous University of Barcelona, Badalona, Spain; Growth and Development Research Group, Vall d’Hebron Research Institute (VHIR), Center for Biomedical Research on Rare Diseases (CIBERER), Instituto de Salud Carlos III, Barcelona, Catalonia, Spain

**Keywords:** 46,XY DSD, biochemical diagnosis, diagnostic algorithm, differences/disorders of sex development (DSD), genetic diagnosis

## Abstract

**Objectives:**

46,XY differences/disorders of sex development (DSD) involve an abnormal gonadal and/or genital (external and/or internal) development caused by lack or incomplete intrauterine virilization, with or without the presence of Müllerian ducts remnants.

**Content:**

Useful biochemical markers for differential diagnosis of 46,XY DSD include hypothalamic-pituitary-gonadal hormones such as luteinizing and follicle-stimulating hormones (LH and FSH; in baseline or after LHRH stimulation conditions), the anti-Müllerian hormone (AMH), inhibin B, insulin-like 3 (INSL3), adrenal and gonadal steroid hormones (including cortisol, aldosterone, testosterone and their precursors, dihydrotestosterone and estradiol) and the pituitary ACTH hormone. Steroid hormones are measured at baseline or after stimulation with ACTH (adrenal hormones) and/or with HCG (gonadal hormones).

**Summary:**

Different patterns of hormone profiles depend on the etiology and the severity of the underlying disorder and the age of the patient at diagnosis. Molecular diagnosis includes detection of gene dosage or copy number variations, analysis of candidate genes or high-throughput DNA sequencing of panels of candidate genes or the whole exome or genome.

**Outlook:**

Differential diagnosis of 46,XX or 46,XY DSD requires a multidisciplinary approach, including patient history and clinical, morphological, imaging, biochemical and genetic data. We propose a diagnostic algorithm suitable for a newborn with DSD that focuses mainly on biochemical and genetic data.

## III. Biochemical and genetic markers in 46,XY differences/disorders of sex development (46,XY DSD)

Biochemical work-up in patients with a 46,XY karyotype and discordances in gonadal and/or genital development should include measurements of the hormones involved in testicular development and an evaluation of androgen and anti-Müllerian hormone (AMH) synthesis and action [1]. The hypothalamus-pituitary-testicular (HPT) axis is very active during fetal development. The follicle-stimulating hormone (FSH) controls the proliferation of Sertoli cells, determining the increase of testicular volume and the secretion of AMH and inhibin B (INHB); the luteinizing hormone (LH) regulates Leydig cells, stimulating the secretion of androgens, mainly testosterone (T), and insulin-like 3 (INSL3), involved in the descent of testes to scrotal pouches [2]. At birth, blood concentrations of T, LH and FSH are low in males, although higher than in females, which progressively increase from the end of the first week, peak by the 3rd month, and thereafter decrease gradually to reach low levels by 6 months, and continue to be low until the onset of puberty [3]. This physiological activation of the HPT axis is called “minipuberty” and is an optimal period to study infants with differences/disorders of sex development (DSD) during the first months of life. From the end of the first semester of life, serum levels of gonadotropins and T are very low; hence, to evaluate the HPT axis or Leydig cell capacity to synthesize T, it is necessary to stimulate the pituitary gland with the gonadoliberin (GnRH) or its analogs, or the testes with chorionic gonadotropin (HCG). However, there are two peptide hormones produced by Sertoli cells (AMH and INHB) that are excellent markers of seminiferous tubule function during infancy, childhood and puberty [4].

AMH is exclusively secreted by Sertoli cells, and is a good marker of primary testicular dysfunction [5]. AMH concentrations are very high in infants and prepubertal children and decrease at onset of puberty, whereas T concentrations increase. AMH concentrations must be interpreted according to age and pubertal stage [6]. AMH concentrations are decreased or even undetectable in patients with gonadal dysgenesis, although they are preserved in patients with interstitial compartment disorders (isolated deficiency of androgen synthesis or action).

INHB is also primarily secreted by Sertoli cells, though not exclusively. It is the main inhibitor of pituitary FSH secretion. Although it does not have a specific role in fetal sex differentiation, it is a useful marker of testicular function. INHB concentrations are low in the newborn and increase throughout the first month of life, peaking at two years. From that moment on, INHB concentrations decrease progressively until the onset of puberty, remaining at low but detectable concentrations during adult life [7, 8].

### 1) Abnormal gonadal development

In 46,XY DSD, abnormal gonadal development may result in partial or complete gonadal dysgenesis (PGD or CGD) or even lead to ovotesticular (testicular and ovarian tissues in the same or separate gonads) or ovarian gonads (Table 1).

**Table 1:** Classification of disorders or differences of sex development (DSD) based on the XY sex chromosomes present in the karyotype.

DSD with 46,XY karyotype	
1. Abnormal gonadal development	a. Partial (PGD) or complete (CGD) gonadal dysgenesis
	b. Ovotesticular DSD
	c. Ovarian DSD
2. Abnormal genital development secondary to deficient androgen synthesis or action	**Disorders of androgen synthesis:**
	a. Insensitivity to LH (Leydig cell aplasia/hypoplasia)
	b. 7-Dehydrocholesterol reductase deficiency (Smith–Lemli–Opitz syndrome)
	c. Star protein deficiency (lipoid congenital adrenal hyperplasia)
	d. Cholesterol desmolase deficiency
	e. 3β-Hydroxy steroid dehydrogenase type 2 deficiency
	f. 17α-Hydroxylase/17-20 desmolase deficiency
	g. P450 oxidoreductase deficiency
	h. Cytochrome b5 deficiency
	i. Defective backdoor steroidogenesis pathway
	j. 17β-Hydroxy steroid dehydrogenase type 3 deficiency
	k. 5α-Reductase type 2 deficiency
	l. Isolated hypospadias and/or cryptorchidism
	**Disorders of androgen action:**
	a. Complete or partial androgen insensitivity
	b. Therapeutic agents or endocrine disrupting contaminants
3. Abnormal genital development secondary to defective anti-Müllerian (AMH) hormone synthesis or action	**Persistent Müller ducts:**
	a. Anti-Müllerian hormone deficiency
	b. Resistance to anti-Müllerian hormone
4. Complex malformation syndromes	a. Malformative syndromes with abnormal genital development (cloacal malformations, Aarskog syndrome, Robinow syndrome, among others)
	b. Severe, early-onset, intrauterine growth retardation

CGD patients present female internal and external genitalia. Bilateral streak gonads develop instead of normal gonads, which are unable to secrete androgens or AMH. At biochemical level, subjects develop a hypergonadotropic hypogonadism with increased LH and FSH concentrations, T deficiency, and undetectable AMH and INHB.

In PGD, the degree of masculinization is determined by the mass of functional testicular tissue. Persistence of Müllerian structures reflects a deficiency of AMH secretion, a sign of Sertoli cell dysfunction.

Thanks to massive sequencing techniques, an increasing number of genes involved in PGD and CGD have been identified (see Table 2). The first gene to be described was *SRY*. Many of these genes are associated with disorders in other tissues or systems (Table 2): *ARX*, *ATRX*, *CDKN1C*, *DHH*, *DMRT1*, *DMRT2*, *EMX2*, *FANCAFGFR2*, *GATA4*, *HHAT*, *LHX9*, *MYRF*, *NR5A1*, *PBX1*, *PPP2R3C*, *SAMD9*, *SOX9*, *TSPYL1*, *WT1.* In others, no associations had been described (Table 2): *DAX1* duplications, dominant mutations in *DHX37*, *MAP3K1*, *WWOX* or *ZNRF3*, recessive mutations in *EFCAB6*, monoallelic or biallelic mutations in *ESR2* or *SOX8*, X-linked mutations in *FTHL17*, *MAMLD1* or *STARD8*.

**Table 2:** Clinical diagnoses and genes involved in disorders or differences of sex development (DSD) of monogenic etiology.

DSD with 46,XY karyotype		
Clinical diagnosis	Gene (locus)	OMIM (inheritance) (additional phenotype)
**1. 46,XY DSD secondary to impaired gonadal development: complete gonadal dysgenesis (CGD) or partial gonadal dysgenesis (PGD), ovotesticular DSD, ovarian DSD**		
PGD	*ARX* (Xp21.3)	300215 (XL:D) (lissencephaly, epilepsy, mental retardation)
PGD	*ATRX* (Xq21.1)	300032 (D: del) (mental retardation, α-thalassemia)
PGD/CGD/ovarian DSD	*CBX2* (17q25.3)	602770 (RA)
		613080 (RA) *CBX2.1*/(DA) *CBX2.2*
PGD/CGD	*CDKN1C* (11p15.4)	600856 (DA)/614732 (IMaGE syndrome: intrauterine growth retardation, metaphyseal dysplasia, congenital adrenal hypoplasia, genital abnormalities)
PGD/CGD	*DAX1* (*NR0B1*) (Xp.21)	300018 (XL:dup)
PGD/CGD	*DHH* (12q13.12)	233420/607080 (RA/DA) (minifascicular neuropathy)
PGD/CGD	*DHX37* (12q24.31)	617362 (DA) (includes testicular regression syndrome)
PGD/CGD	*DMRT1* (9p24.3)	602424 (DA:del) (with or without mental retardation)
	*DMRT2* (9p24.3)	604935 (DA:del) (with or without mental retardation)
CGD	*EFCAB6* (22q13.2-q13.31)	64800 (RA)
PGD	*EMX2* (10q26.11)	600035 (DA:del) (mental retardation, renal agenesis)
PGD/CGD	*ESR2* (14q23.2-q23.3)	601663 (biallelic and monoallelic)
PGD	*FANCA* (16q24.3)	606139 (RA) (Fanconi anemia and microcephaly)
PGD/CGD	*FGFR2* (10q26.13)	176943 (DA) (craniosynostosis) (only a case reported)
CGD	*FTHL17* (Xp21.2)	300308 (XL) (family tree)
PGD/CGD	*GATA4* (8p23.1)	615542/600576 (DA) (with or without congenital heart disease)
CGD	*HHAT* (1q32.2)	605743 (RA) (only a family reported: short stature, generalized chondrodysplasia, muscle hypertrophy, myopia, mild mental retardation)
CGD	*LHX9* (1q31.3)	606066 (DA) (with lower limb malformations)
PGD	*MAMLD1* (*CXOrf6*) (Xq28)	300120 (XL) (X-linked hypospadias)
PGD/CGD	*MAP3K1* (*MEKK1*) (5q11.2)	613762/600692 (DA)
GD	*MYRF* (11q12.2)	608329 (DA)/618280 (DA) (urogenital cardiac syndrome)
PGD/CGD	*NR5A1* (9q33.3)	612965/184757 (DA)/(RA) (primary adrenal insufficiency/hypogonadotropic hypogonadism, very rare)
PGD	*PBX1* (1q23.3)	176310 (DA) (pulmonary hypoplasia, hypotonia, psychomotor retardation, deafness ± ear abnormality, nephrourological abnormalities, growth retardation)
CGD	*PPP2R3C* (14q13.2)	618419 (RA)/618420 (DA) (dysmorphic facies, retinal dystrophy and myopathy; spermatogenesis failure)
GD	*SAMD9* (7q21.2)	610456 (DA)/617053
		MIRAGE syndrome (Myelodysplasia, Infections, growth Retardation, Adrenal hypoplasia, Genital phenotype, Enteropathy)
PGD/CGD	*SOX8* (16p13.3)	605923
PGD/CGD	*SOX9* (17q24.3)	114290 (AD) (campomelic dysplasia)
PGD/CGD	*SRY* (Yp11.2)	400044/480000 (D)
PGD/CGD	*STARD8* (Xq13.1)	300689 (XL)
PGD/CGD	*TSPYL1* (6q22.1)	604714/608800/(RA) (SIDDT “Sudden Infant Death with Dysgenesis of the Testes” syndrome)
CGD/ovotesticular DSD/ovarian DSD	*WNT4* (1p36.12)	603490 (DA: dup)
PGD	*WT1* (11p.13)	607102 (DA)
		(1) 194072 (Del 11.p13) (WAGR syndrome: Wilms tumor, psychomotor Retardation and Aniridia)
		(2) 194080 (inactivation) (Denys-Drash syndrome: nephropathy, Wilms tumor, risk of gonadoblastoma)
		(3) 136680 (splice-site) (Frasier syndrome: kidney disease, high risk of gonadoblastoma)
PGD	*WWOX* (16q23.1-q23.2)	605131 (DA:del) (only a reported case of maternal inheritance)
PGD/CGD/ovotesticular DSD	*ZFPM2* (*FOG2*) (8q23.1)	616067/603693 (DA) (with or without congenital heart disease)
PGD/CGD	*ZNRF3* (22q12.1)	612062 (DA)
**2. 46,XY DSD with impaired androgen biosynthesis or activity, isolated hypospadias, isolated cryptorchidism**		
Insensitivity to LH/Chorionic gonadotropin	*LHCGR* (2p16.3)	238320 (RA) (Leydig cell aplasia/hypoplasia)
7-Dehydrocholesterol reductase deficiency	*DHCR7* (11q13.4)	270400 (RA) (Smith–Lemli–Opitz syndrome)
STAR deficiency (lipoid CAH)	*STAR* (8p11.23)	201710 (RA)
(1) Classical form		(1) Adrenal and gonadal deficiency

(2) Non-classical form		(2) Adrenal deficiency

CAH secondary to cholesterol desmolase deficiency	*CYP11A1* (15q24.1)	613743 (RA) (adrenal and gonadal deficiency)
CAH secondary to 3β-hydroxy steroid dehydrogenase type 2 deficiency	*HSD3B2* (1p12)	201810 (RA) (adrenal and gonadal deficiency)
17α-Hydroxylase/17,20 desmolase deficiency	*CYP17A1* (10q24.32)	202110 (RA)
(1) CAH secondary to combined deficiency		(1) CAH + hypertension + gonadal deficiency
(2) Isolated 17,20 desmolase deficiency		(2) Gonadal deficiency
P450-oxidoreductase deficiency	*POR* (7q11.23)	201750 (RA) (variable deficiencies of 17α-hydroxylase, 21-hydroxylase and aromatase); (Antley-Bixler syndrome, ± craniosynostosis)
Cytochrome b5 deficiency	*CYB5A* (18q22.3)	250790 (RA) (methemoglobinemia type IV)
Defective backdoor steroidogenesis pathway	*AKR1C2* (10p15.1)	614279 (RA)
	*AKR1C4* (10p15.1)	DSD secondary to DHT deficiency with apparent 17,20-desmolase deficiency and normal *CYP17A1* and *SRD5A2* genes
17β-Hydroxysteroid dehydrogenase type 3 (17-keto-reductase) deficiency	*HSD17B3* (9q22.32)	264300 (RA) (gonadal deficiency)
5α-reductase type 2 deficiency	*SRD5A2* (2p23.1)	264600 (RA)
X-linked hypospadias	*MAMLD1* (*CXOrf6*) (Xq28)	300758 (XL) (hypospadias)
Isolated hypospadias	*ATF3* (1q32.3)	603148 (DA ??)
Cryptorchidism	*INSL3* (19p13.11)	219050 (DA)
Cryptorchidism	*RXFP2* (*LGR8/GREAT/GPR106*) (13q13.1)	606655 (DA ??)
Complete or partial androgen insensitivity	*AR* (Xq12)	300068/312300/300633 (XL)
**3. 46,XY DSD secondary to defective anti-Müllerian hormone (AMH) secretion or activity**		
AMH deficiency	*AMH* (19p13.3)	261550 (RA) (persistency of Müller ducts type I: inguinal uterine hernia and bilateral cryptorchidism)
Insensitivity to AMH	*AMHR2* (12q13.13)	261550 (RA) (persistency of Müllerian ducts type II: inguinal uterine hernia and bilateral cryptorchidism)

DSD, disorders or differences of sex development; GD, gonadal dysgenesis; PGD, partial gonadal dysgenesis; CGD, complete gonadal dysgenesis; CAH, congenital adrenal hyperplasia ; D, dominant; DA, dominant autosomal; RA, recessive autosomal; XL, X-linked; T, translocation; Dup, duplication; Del, deletion; ??, unknown.

Ovotesticular or ovarian development is very rare, and diagnosis is based on anatomopathological analysis. Genetic markers include duplications of *WNT4* gene (3 copies) and monoallelic mutations in *CBX2* or *ZFPM2* (*FOG2*) genes (Table 2). The three have also been identified in patients with CGD or PGD.

### 2) Abnormal genital development secondary to deficient androgen synthesis or action

In 46,XY DSD patients with testicular gonads, masculinization of the internal and external genitalia may be deficient or absent due to disorders of androgen biosynthesis or action (Table 1).

#### a) Disorders of androgen biosynthesis

Isolated deficiency of androgen biosynthesis along with a normal testicular development includes a deficient T production by Leydig cells [LH resistance]; deficient T biosynthesis [Table 1(2-a–j)]; and dihydrotestosterone (DHT) deficiency secondary to 5α-reductase type 2 deficiency [Table 1(2-k)]. In all cases, the uterus is absent, as the testes secrete AMH at normal levels. Several deficiencies in T testicular biosynthesis may also affect the adrenal biosynthesis [9].

##### a-1) Lack of sensitivity to LH (Leydig cell aplasia/hypoplasia). LH receptor disorders

The decreased or lack of expression of gonadotropin LH and HCG receptor in the testicular interstitial cells, which are the precursors of Leydig cells, gives rise to Leydig cell hypoplasia or complete agenesis. T biosynthesis is absent or reduced and cannot be stimulated. The external genitalia can be completely female or ambiguous, with hypospadias and cryptorchidism. The biochemical profile includes elevated baseline LH concentrations and increased response to GnRH or its analogs, concurrent with normal or non-increased FSH concentrations. T concentrations are low and do not increase under HCG stimulation, with all T precursors being normal. AMH and INHB concentrations are within the normal range for age, as Sertoli cell function and response to FSH are both normal [10, 11].

Molecular diagnosis is based on the detection of biallelic mutations in the *LHCGR* gene (Table 2).

##### a-2) 7-Dehydrocholesterol reductase deficiency (Smith–Lemli–Opitz syndrome)

In the Smith–Lemli–Opitz syndrome (SLO), 7-dehydro-cholesterol reductase deficiency severely impairs cholesterol metabolism, which results in very low concentrations of cholesterol in plasma and significantly increased concentrations of the precursor 7-dehydrocholesterol. Since cholesterol is essential in multiple biological processes, patients present a broad spectrum of congenital malformations and intellectual deficit including growth and mental retardation, microcephaly, characteristic facial traits, syndactyly of the 2nd and 3rd toes and cardiovascular anomalies, among other features. In addition, as cholesterol is the precursor of all steroid hormones, patients have adrenal insufficiency, androgen deficiency and genital ambiguity, with micropenis, hypospadias, cryptorchidism and bifid scrotum, which are accompanied by other anomalies of the urinary tract and kidneys [12].

Molecular diagnosis includes the presence of biallelic mutations in *DHCR* gene (Table 2).

##### a-3) StAR protein deficiency (lipoid congenital adrenal hyperplasia)

StAR protein (steroidogenic acute regulatory protein) regulates the transfer of cholesterol from the outer to the inner mitochondrial membrane which is the rate-determining step of adrenal and gonadal steroidogenesis. Patients with StAR deficiency present severe congenital adrenal hyperplasia (CAH), with deficiency of glucocorticoids and mineralocorticoids, clinically manifested by hypoglycemia, hyperpigmentation of skin and mucosa, dehydration and shock. Biochemical disorders include deficiency of cortisol, aldosterone and their precursors, elevated levels of adrenocorticotropic hormone (ACTH) and plasma renin activity (PRA), hyponatremia and hyperkalemia. In 46,XY male infants, deficiency of testicular androgens is associated with different degrees of genital ambiguity, with micropenis, hypospadias, and cryptorchidism. T and its precursors are decreased and do not respond to HCG stimulation, whereas LH concentrations are increased. Impaired cholesterol metabolism causes the accumulation of lipid drops in the adrenal gland tissue (lipoid CAH) and in the testis interstitium [13].

Classic forms exhibit adrenal and gonadal deficiency, whereas milder or non-classic forms are characterized by isolated adrenal deficiency, although gonadal deficiency may develop after puberty [14]. All patients display biallelic mutations in the *StAR* gene (Table 2).

##### a-4) Cholesterol desmolase deficiency

The mitochondrial enzyme cholesterol 20-22-desmolase (P450 *side chain cleavage*) catalyzes the conversion of cholesterol into pregnenolone, a rate-determining step of adrenal and gonadal steroidogenesis. The clinical and biochemical effects of this deficiency mimic those of StAR deficiency. Differential diagnosis is based on genetic testing showing biallelic mutations in *CYP11A1* gene (Table 2). In this case, alterations occur both at adrenal and gonadal level.

##### a-5) 3β-Hydroxysteroid dehydrogenase type 2 (HSD3B2) deficiency

Enzyme HSD3B2 catalyzes the conversion of delta 5 steroids (Δ5) [pregnenolone, 17α-hydroxy-pregnenolone (17OH-Preg) and dehydroepiandrosterone (DHEA)] to delta 4 (Δ4) steroids. Severe forms have a clinical presentation similar to that of StAR or cholesterol desmolase deficiency. Patients with milder forms may not display mineralocorticoid deficiency or clinical signs of salt wasting. It is associated with ambiguous genitalia secondary to testicular T deficiency. Specific diagnosis relies on increased levels of basal 17OH-Preg and DHEA or after ACTH stimulation; DHEA sulphate (DHEA-S) concentrations are also elevated. Δ4 steroid [17α-hydroxyprogesterone (17OH-P) and androstendione] concentrations should be decreased, but instead they can be elevated by the peripheral action of the iso-enzyme 3β-hydroxysteroid dehydrogenase type 1 (HSD3B1), thus challenging differential diagnosis with 21-hydroxylase and 11β-hydroxylase deficiencies. Since 21-hydroxylase deficiency is a much more prevalent disorder, differential diagnosis often includes testing both *CYP21A2* and *HSD3B2* genes, although in 46,XY patients, genital ambiguity is more suggestive of HSD3B2 deficiency. Mutations in *HSD3B2* gene are biallelic (Table 2).

##### a-6) 17α-Hydroxylase/17-20 lyase or desmolase deficiency

Microsomal enzyme steroid 17α-hydroxylase/17,20-lyase (also CYP17 or P450C17) is expressed in the adrenal cortex and the gonads. 17α-hydroxylation catalyzes the conversion of pregnenolone to 17OH-Preg and from progesterone (P) to 17OH-P. Deficiency of this enzyme blocks the synthesis of cortisol, androgens, and estrogens. The activity of 17-20 lyase or desmolase catalyzes the conversion of 17OH-Preg to DHEA and of 17OH-P to androstendione, although the last step is much less efficient [15, 16].

In combined 17α-hydroxylase and 17-20 lyase deficiencies, increased levels of ACTH induced by cortisol deficiency causes a significant increase of pregnenolone, P, and 11-deoxycorticosterone, which has a mineralocorticoid activity. As a result, clinical signs of mineralocorticoid excess such as arterial hypertension, alkalosis, hypokalemia, and PRA suppression associate to those of CAH. Male 46,XY infants lack masculinization of the external and internal genitalia, display hypoplasia of structures derived from Wolff ducts and testicular gonads are intraabdominal. Combined 17α-hydroxylase and 17-20 lyase deficiency is the result of biallelic mutations with severe inactivation effects in the *CYP17A1* gene (Table 2). Isolated 17-20 lyase deficiency only affects gonadal steroidogenesis; therefore, the DSD is induced by testicular T deficiency, with decreased levels of androstenedione and T and normal or slightly elevated levels of P and 17OH-P. This isolated form is caused by less severe mutations of the gene, with the enzyme function being only partially inactivated (Table 2).

##### a-7) P450-oxidoreductase (POR)

P450-oxidoreductase (POR) deficiency can also be the cause of a CAH in 46,XY patients and lead to genital ambiguity caused by a deficient production of T by the gonads. Patients may exhibit a combined deficiency of the enzymatic activities mediated by this electron donor [CYP17, 21-hydroxylase (CYP21) and aromatase (CYP19)]. These activities may be disrupted to different degrees, depending on mutations in the *POR* gene [17]. CYP21 deficiency manifests by increased serum concentrations of 17OH-P and a higher response to ACTH stimulation. However, low CYP17 activity may mask CYP21 deficiency and cause basal T and androstenedione deficiency, which may be detected either during minipuberty or prepuberty after HCG stimulation. Molecular diagnosis should evidence the presence of biallelic mutations in *POR* gene (Table 2).

##### a-8) Cytochrome b5 deficiency

Cytochrome b5 (cyb5) is a small hemoprotein that modulates the enzyme CYP17. It is expressed in the fasciculate and reticular areas of the adrenal cortex and in the gonads. CYP17 catalyzes two reactions in the steroidogenic pathway (17α-hydroxylation and 7,20-desmolase), and cyb5 facilitates the activity of 17,20-lyase reaction [16]. Male 46,XY infants present genital ambiguity, with deficient masculinization and methemoglobinemia [18]. The biochemical profile corresponds to isolated 17,20-lyase deficiency, decreased levels of T and its precursors (DHEA and androstenedione), increased concentrations of 17OH-P and 17OH-Preg. In the HCG stimulation test, T does not increase, and the ratio of C21 steroids (deoxycortisol) to C19 steroids (DHEA and androstenedione) is increased. Gonadotropin concentrations are elevated. Molecular diagnosis relies on the presence of biallelic mutations in the *CYB5A* gene (Table 2).

##### a-9) Backdoor steroidogenesis pathway deficiency

Enzymes of the aldoreductase family (AKR1C2 and AKR1C4) are involved in P and 17OH-P processing to DHT through the “backdoor” pathway overcoming T synthesis. There are reports of families in which 46,XY members display genital ambiguity and a phenotype similar to that of 5α-reductase type 2 or CYP17 deficiency with isolated deficiency of 17,20-lyase activity. These subjects exhibit inactivating mutations in *AKR1C2* and *AKR1C4* (Table 2). This finding indicates that fetal DHT production through the alternative pathway, without T mediation, is also necessary for complete virilization of the external genitalia [19]. The role of molecular defects in this steroidogenesis pathway has also been questioned in the genesis of hypospadias without other clinical or biochemical characteristics [20].

##### a-10) 17β-Hydroxysteroid dehydrogenase (17B-HSD) type 3 deficiency

The enzyme 17B-HSD catalyzes 17β-oxidation and the reduction of steroids C18 and C19 in several tissues. Several isoenzymes have been described, and type 3 is the one expressed in the testes, where it catalyzes the conversion of androstenedione to T, DHEA to 5-androstenediol and estrone (E1) to estradiol (E2) [21, 22]. Patients with deficiency of this enzyme present genital ambiguity ranging from less severe degrees to completely female genital. At biochemical level, patients present very high concentrations of androstenedione with normal or low T, which results in a very high androstenedione/T ratio, both at baseline and after HCG stimulation. DHEA, DHT, E1 and E2 may also be elevated. In puberty, gonadotropins concentrations increase [23]. Molecular diagnosis relies on the presence of biallelic mutations in the *HSD17B3* gene (Table 2).

##### a-11) 5α-Reductase type 2 deficiency

This enzyme catalyzes the transformation of T to DHT, which is the most powerful androgen (it binds the same androgen receptor (AR), but with 10 times more affinity). DHT is responsible for the differentiation of the urogenital sinus and genital tubercle into a male system (prostate, corpus cavernosum, male urethra and scrotal sacs) [24]. Male 46,XY infants with a deficiency of this enzyme exhibit almost female genitalia at birth and are often identified as female. However, during puberty, T production increases causing a striking virilization of the external genitalia, the growth of a penis and the appearance of secondary male sexual characteristics, with an increase of muscle mass, voice masculinization and increased libido, except for the scarce growth of sexual hair.

At biochemical level, they have normal T concentrations, with an increased T/DHT ratio at baseline or after HCG stimulation. Standard cut-off values for this ratio cannot be well established as it varies considerably in normal children and changes with age (higher in breastfed infants) and with puberty. Some authors have suggested cut-off points for T/DHT > 25 in prepuberty. However, lower levels have been reported elsewhere (with a mean of 12 in patients with mutations in the *SRD5A2* gene) [25]. This ratio may also be elevated in patients with lack of sensitivity to androgens (due to a relative deficiency in this enzymatic activity), which is usually a source of false positive diagnosis [26, 27]. In addition, the DHT immunoassays currently available have low sensitivity and specificity, and this ratio should therefore not be used for differential diagnosis. Measurement of urinary glucocorticoid and androgen metabolites by tandem gas chromatography-mass spectrometry (GC-MS/MS) provides the best diagnostic sensitivity and specificity for this disorder, both in prepubertal children and in gonadectomized adults: the decrease in the ratio of the 5α- to 5β-reduced metabolites (androsterone/etiocholanolone, in the case of androgens) is pathognomonic for type 2 5α-reductase deficiency [24, 28].

Diagnosis must be confirmed by demonstrating the presence of biallelic mutations in the *SRD5A2* gene (Table 2) [29].

##### a-12) Isolated hipospadias and/or cryptorchidism

The search for the genes involved in DSDs with a 46,XY karyotype when the candidate genes previously described are normal has prompted the description of several genes which mutations seem to be associated with the phenotype. Among them, the most frequently described genes include (Table 2):

– *MAMLD1* (*CXOrf6*) located in chromosome X, explaining why some authors refer to this phenotype as “X-linked hypospadias” [30, 31]. The pathogenicity of mutations has also been a subject of debate. Some authors state that phenotypes not showing a specific set of biomarkers could be the result of the combination of mutations in several genes, the separate effects of which may not have phenotypical consequences [32, 33].
– *ATF3* gene encodes a transcription factor that activates the androgen receptor (AR) gene promoter. These mutations have a dominant effect [34].
– *INSL3* gene encodes the INSL3 protein (insulin-like protein 3), which regulates the descent of the testes to the scrotal pouches during the third trimester of the fetal development. Monoallelic mutations have been described in families with isolated cryptorchidism [35].
– *RXFP2*, the INSL3 receptor gene (also known as *LGR8/GREAT/GPR106*) has also been associated with isolated cryptorchidism [34].

#### b) Impaired androgen action

The syndrome of androgen insensitivity is a recessive X-linked disorder that affects the androgen receptor. It is the most frequent cause of 46,XY DSD with female phenotype [36]. In the complete form (complete androgen insensitivity syndrome, CAIS), patients present completely female external genitalia, male internal genitalia, except for a short vagina (some patients may present a uterus) and normally developed, although intra-abdominal, testes that may have been identified as bilateral inguinal hernias. Sertoli and Leydig cell activities are preserved, and T and AMH concentrations are either normal or elevated [37]. At prepuberty, baseline T is low, as it corresponds with this stage of development, but T response to HCG stimulation is similar to that in healthy male children or even in the upper limit of normality [36]. At puberty, LH concentration increases, with normal or slightly elevated FSH, and levels of E2 and sex hormone-binding globulin (SHBG) are elevated for the male sex.

Partial androgen insensitivity syndrome (PAIS) displays different degrees of sexual ambiguity (hypospadias, micropenis, bifid scrotum) and a less identifiable biochemical profile. LH and T are generally elevated during the first months of life (minipuberty), as it occurs in healthy males, whereas this is not always observed in CAIS [38].

Diagnosis of CAIS or PAIS requires molecular confirmation and the identification of a mutation in the *AR* gene (Table 2). The association between clinical and biochemical phenotypes and molecular diagnosis is very high in CAIS, whereas in PAIS the percentage of molecular diagnoses is very low [39]. In some cases, the mutation in the *AR* has been proven not to be germinal, but acquired, postzygotic, presenting cellular mosaicism [40].

There are patients with the PAIS phenotype that some authors call “idiopathic” since it is not associated with any genetic alteration. In some cases, it has been possible to demonstrate that the cause of this phenotype was fetal exposure to therapeutic agents administered to the mother (estrogens, including clomiphene) or to environmental pollutants that exert anti-androgenic actions [41], [42], [43], [44].

#### c) Abnormal genital development secondary to abnormal AMH secretion or activity

The isolated persistence of Müllerian ducts can be due to isolated AMH deficiency due to *AMH* gene inactivating mutations or to insensitivity to AMH due to mutations inactivating the gene that encodes its receptor (*AMHR2*) [45] (Table 2). The two syndromes share a similar clinical picture. These two syndromes differ in that in *AMH* gene mutations, serum AMH concentrations are undetectable, whereas in AMH receptor mutations, AMH concentrations are normal or elevated. Persistence of Müllerian ducts is also associated with gonadal dysgenesis, in which there is an alteration of testicular development (with involvement of Leydig and Sertoli cells), so differential diagnosis should involve histopathological, biochemical and molecular testing.

#### d) Complex malformation syndromes

Some of the gene mutations causing gonadal dysgenesis and consequently 46,XY DSD are also associated with malformations in other tissues and systems leading to different malformation syndromes (*SOX9*, *WNT*, *WT1*, etc.) (Table 2). Numerous genetic structural and monogenic alterations linked to complex malformation syndromes may also cause renal and cardiac disorders, growth and mental retardation, and involve gonadal and/or genital malformations [46]. These syndromes include Aarskog syndrome, Robinow syndrome, and cloacal anomalies (Table 1).

Finally, it is worth mentioning that 46,XY fetuses with early-onset and severe intrauterine growth retardation (Table 1) present hypospadias more frequently than children with adequate weight for gestational age. In these patients, if genetic causes have been ruled out, an abnormality in the chronology of sexual development has been proposed as the cause of the hypospadias [47].

## IV) Proposals for the differential diagnosis of DSD focused on biochemistry data and genetic workup

The differential diagnosis of a patient with DSD must have a multidisciplinary approach, involving several medical specialties. The team has to be coordinated by a clinician who, depending on the patient’s age and the reason for referral, can be a neonatologist, a pediatrician or an endocrinologist. The biochemistry workup will encompass general analytics, protein and steroids hormones in blood and/or urine samples obtained in baseline conditions or in the context of dynamic stimulation tests according to the patient’s age (newborn, infant, prepubertal child, adolescent or adult), the accompanying clinical features and the karyotype.

Genetic testing starts with a karyotype followed by molecular testing if a monogenic etiology is suspected. Numerous diagnostic algorithms have been proposed, based on the karyotype, presence or not of palpable gonads, abdominal ultrasonography and the hormones tested [48], [49], [50], [51], [52].

Presumptive molecular diagnosis should be established considering the information obtained from the different studies performed. Massive high-throughput sequencing techniques (candidate gene panels or whole exome) provide a rapid first approach to the most frequently known causes. Also, subsequent revision of results enables the analysis of other less frequent or novel causes [53], [54], [55], [56], [57].

**Figure 1:**
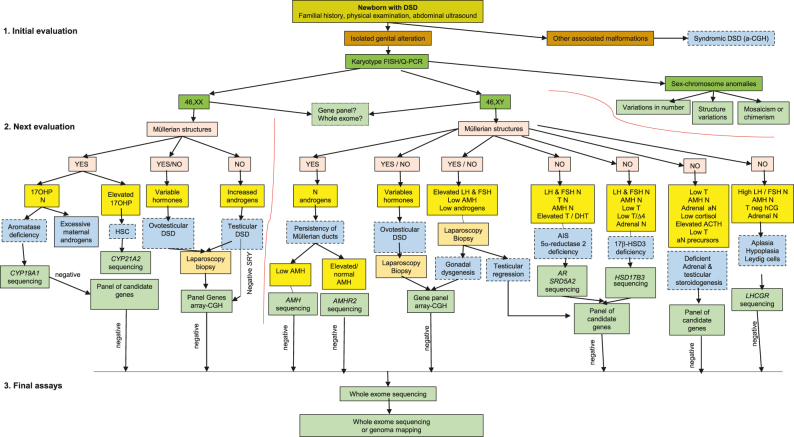
Diagnostic algorithm for a newborn or breastfed infant with a different sexual development (DSD) due to a discordance between levels of female or male sexual differentiation (genetic and genital sex). Initial evaluation will include familial history, physical examination and an abdominal ultrasound. If the patient shows other malformations added to genital malformations, a syndromic DSD is established, and a comparative genomic hybridation array (a-CGH), followed by other genetic tests, is performed. If the subject only shows an isolated genital anomaly, the karyotype (obtained by cytogenetic or more rapidly by a-CGH; also the detection of chromosomes X and Y centromeres and of the *SRY* gene by fluorescent *in situ* hybridization (FISH) or quantitative PCR (Q-PCR)] subclassifies DSD into one of the following three diagnostic groups: 46,XX, 46,XY or sexual chromosome anomalies. This last group is subclassified relying on whether variation is quantitative, structural or the presence or not of mosaicism. Next, evaluation continues in 46,XX and 46,XY patients, based on abdominal ultrasound findings (presence or not of Müllerian structures) and hormone determination results, which provide diagnostic guidance and, as a result, the panel of candidate genes to be sequenced. Genes can be either analyzed separately, or by massive sequencing of panels of candidate genes, or by whole exome or whole genome sequencing. A CGH, array-complementary genome hybridization; FISH, fluorescent *in situ* hybridization; Q-PCR, quantitative polymerase chain reaction; DSD, different sexual development; 17OH-P, 17-hydroxy-progesterone; N, normal; aN, abnormal; LH, luteinizing hormone; FSH, follicle stimulating hormone; AMH, anti-Müllerian hormone; T, testosterone; DHT, dihydrotestosterone; D4, androstenedione; ACTH, adrenocorticotropic hormone; T neg HCG, lack of response of T to the hCG test; CAH, congenital adrenal hyperplasia; AR, androgen receptor; AIS, androgen insensitivity syndrome. *SRY*, sex-determining region of the Y chromosome gene; *CYP19A1*, aromatase gene; *LHCGR*, LH/chorionic gonadotropin receptor gene; HSD17B3, 17 beta-hydroxysteroid dehydrogenase 3 gene; *CYP 21*, A2, 21 hydroxylase gene; *SRD5A2*, steroid 5 alpha-reductase 2 gene.

We propose a diagnostic algorithm suitable for a newborn with DSD mainly based on biochemical and genetic data (Figure 1).

Initial evaluation will include familial history, physical examination and an ultrasonography of the abdomen.

If the patient shows other malformations added to genital anomalies, a syndromic DSD is established, and a comparative genomic hybridisation array (a-CGH) will be performed, followed by other genetic tests.

When the genital malformation is isolated, the karyotype subclassifies the DSD into one of the three diagnostic groups: 46,XX, 46,XY or sex chromosome anomalies. This last group is subclassified relying on whether variation is quantitative, structural or on the presence or not of mosaicism.

The data provided by the abdominal ultrasonography (presence of Müllerian structures), hormone testing, and laparoscopic biopsy will help identify the candidate genes to be tested individually, by massive sequencing of candidate genes, of whole exome or genome.
